# Frostbite Injury of Hand Caused by Liquid Helium: A Case Report

**Published:** 2010-05-19

**Authors:** Celalettin Sever, Yalcin Kulahci, Ali Acar, Haluk Duman

**Affiliations:** ^a^Departments of Plastic and Reconstructive Surgery and Burn Unit; ^b^Infectious Diseases and Clinic Microbiology, Gülhane Military Medical Academy, Haydarpasa Training Hospital, Istanbul, Turkey

## Abstract

**Objective:** The aim of this study was to present a case report of a patient with hand frostbite injury sustained by helium vapor and discuss the circumstances of this injury, treatment, and preventive measures. **Methods:** A case report of the incident was drafted and the relevant literatures were reviewed. The patient was treated with antiedema therapy, extremity elevation, high-molecular-weight dextran, heparin infusion, and hyperbaric oxygen therapy. **Results:** The frostbite injury healed with spontaneous epithelization. At his last follow-up at the eighth month, he had a good range of movement of his hand. **Conclusion:** Frostbite injuries are relatively uncommon and have various etiologies. The adjunctive hyperbaric oxygen therapy is an alternative treatment of frostbite injuries, although it is still considered investigational.

Helium is the second-most abundant element in the universe, after hydrogen. Helium is mostly recovered from natural gas, so it is not a renewable resource. It is a colorless and odorless gas. It is nonflammable and is only slightly soluble in water. When shipped as a liquid, it is very cold and will solidify all other gases. The demand for helium is increasing. Many industries use it, including science and healthcare, metal industry, electronics, and aviation. Helium is the only substance known that does not become solid at temperatures approaching absolute zero at atmospheric pressure.

It is primarily used for cooling metallic superconductors in research and in energy, transport, and telecommunications engineering, as well as in analysis and in medicine. Many metals and alloys completely lose their electrical resistance and become superconductors at liquid helium temperatures. This has led to the development of superconducting magnets that produce intense magnetic fields at low energy cost. These magnets are used in magnetic resonance imaging, an important diagnostic imaging technique that relies on magnetic fields to produce cross-sectional images of the human anatomy.

Liquid helium, the coldest known fluid, is the most important coolant in cryogenics. When liquefied at the cryogenic temperature of -269°C (ie, just above absolute zero), liquid helium causes injuries because of the rapid and profound cooling of the surrounding air, leading to the localized cold injury and cellular destruction.^1^ The numbness develops because of inactivation of nerve sensation. Moreover, skin contact with liquid helium may cause dry skin, contact dermatitis, and mild skin irritation with discomfort or rash. This liquid may also cause severe frostbite. Frostbite following exposure to cold liquids is an occupational hazard.

Up to now, despite its widespread use, a few frostbite cases due to liquid helium have been reported in the literature.^1^,^2^ Most of these injuries may be prevented by taking adequate precautions and educating the persons involved. The aim of this article is to record and investigate a case in which hand frostbite injury was caused by liquid helium.

## CASE REPORT

A 37-year-old man of good general health admitted to our burn care unit with frostbite on his right hand. According to him, the frostbite injury occurred because of contact with helium gas while he was transferring liquid helium from a storage Dewar to the magnet system Dewar. During the transfer, there was a leak of helium from the helium gas container. The right hand was exposed to the gas for about 60 seconds. Immediately after the exposure, the skin had gone white and cold. Afterward, he had felt numbness and increasing pain on his hand.

**Figure 1:**
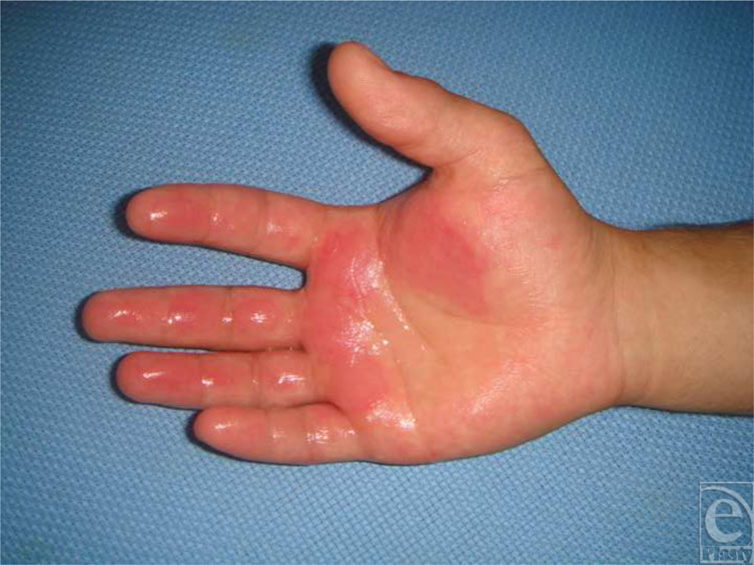
Appearance of frostbite burn caused by helium gas.

Upon arrival at our hospital, the patient was immediately taken to a cleaning tank. His hand was irrigated by 40°C heated and sterilized water for 30 minutes. The initial physical examination demonstrated second- and third-degree frostbite injuries on the palm and fingers (Fig 1) The capillary circulation was clinically adequate. All the fingers were well perfused. Neurological examination showed no motor or sensory response missing.

**Figure 2:**
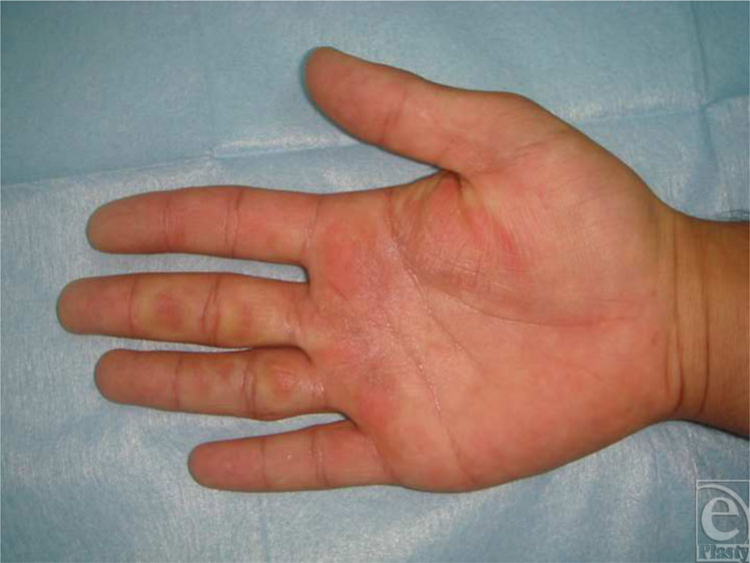
Sixty hours after the injury.

The patient was hospitalized immediately to closely monitor the perfusion. Perfusion of digital fingers was monitored. After 60 hours, the capillary circulation of right hand fingers was poor on inspection (Fig 2). However, there was no black/deep purple discoloration of digits and there was no sign of circulatory compromise assessed by Doppler ultrasonography. Antiedema treatment, extremity elevation, and high-molecular-weight dextran in saline (35 mL/h) were initiated. Heparin infusion (5000 units IV started and 1000 units/h) was used. In addition, we decided to perform hyperbaric oxygen therapy (HBOT), which improves microcirculation, since vein and capillary thromboses are the main causes of tissue damage. Hyperbaric oxygen therapy was repeated daily for 14 days according to the Marx-schema for problem wounds (2, 4 bar, total time at depth = 90 minutes, alternations of 100% O_2_, and air breathing). After the first course of the therapy, we observed rewarming of the injured fingers and pink color at the edges of the affected parts. Remarkable improvement in the frostbite area was seen in daily consultation. On the ninth day, heparin was tapered off and discontinued. Suitable antibiotic therapy was initiated with the consultation of the Infectious Disease Department. Dressings of the burned areas were changed every day. Range-of-motion exercises were performed to prevent contractures. The burns healed with spontaneous epithelization over 4 weeks. During his last follow-up at eighth month, he had a good range of movement of his right hand.

## DISCUSSION

The compressed gases may cause frostbites when they are sprayed on the skin. Contact frostbites are common in young workers and typically involve the hands.^3^,^4^ The upper extremities, the fingers in particular, are more susceptible to cryogenic gas exposure because the vascular structures are smaller and narrower and the tissue coverage is thinner in the upper extremities than in the lower extremities.^5^

Helium gas is not toxic at normal temperature and pressure. Short-time exposure to helium gas may occur via inhalation and skin contact. Helium gas is a simple asphyxiant. Inhalation of this gas will result in unconsciousness, sleepiness, fatigue, loss of coordination, central nervous system depression, respiratory collapse, and death.^1^,^6^ Otherwise, irritation of the eyes, nose, throat, and skin may occur. The repeated skin contact may cause dermatitis.^3^,^6^ Direct contact with liquid helium or prolonged exposure to the chilled gas may produce frostbite. Symptoms of mild frostbite include numbness, prickling, and itching of the affected area. Symptoms of more severe frostbite include a burning sensation and stiffness of the affected area. The skin may become waxy white or yellow. There may be no pain experienced at first, but there is intense pain when the frozen tissue thaws.^6^

The extent of injury caused by helium gas may be determined by the surface area of exposed tissue, the volume of helium gas on the skin, and the duration of exposure. The mechanism of helium gas is unlike frostbite, in that the damage occurs within seconds. However, there is some controversy over the duration of exposure time that may lead to such severe tissue damage. The appearance of the superficial tissue is often an unreliable indicator of the viability of the underlying tissue.^3^,^4^ The injury may be more severe than that caused by a thermal burn because the agent rapidly and deeply penetrates the skin. Because of this reason, frost gas on the skin may also make it more difficult to estimate the extent of the injury.

The pathophysiology of frostbite is thought to have 2 distinct mechanisms: direct cellular damage at the time of exposure to the cold; and postthaw arterial vasoconstriction, leading to disordered vascular flow patterns and damage to the microcirculation. The intracellular electrolyte concentration increases dramatically. This leads to intracellular dehydration with an increase in intracellular electrolytes, proteins, and enzymes that lead to cell death. Generation of oxygen free radicals, production of prostaglandins and thromboxane A_2_, release of proteolytic enzymes, and generalized inflammation are the underlying mechanical effects of these injuries. In addition, vascular endothelial damage leads to intravascular thrombosis and reduced blood flow. The outcome is vascular thrombosis and dermal necrosis.^7^ The treatment of frostbite is directed toward reversing the pathologic effects of ice-crystal formation, vasoconstriction, and the release of inflammatory mediators; therefore, rapid rewarming and anti-inflammatory agents are still the main components of treatment protocols.

There is little information available in the medical literature that report on emergency treatments of hand frostbite injuries. The prophylactic treatment of infection and thrombosis, prevention of compartment syndrome, care of burned areas, and surgical debridement play a significant role in the treatment of frostbites. The first treatment step is to remove the patient from danger and minimize the duration of exposure. Unlike the thermal burn, the agent will continue to damage the tissue until the substance is inactivated or removed from the area. In the first aid treatment, the contaminated skin must be promptly washed using soap or mild detergent and water. If it soaks through the clothing, the clothing must be removed immediately and the underlying skin must be washed as described previously in the text. For frostbite, immediate rewarming in a water bath between 40°C to 42°C is recommended.^4^,^8^ Heparinization for the prevention of thrombosis is still controversial. In the first few days after thawing, thrombosis was seen in the superficial dermal plexus. For this reason, heparinization has been used to prevent intravascular thrombosis.

Best frostbite treatment results have been achieved with methods that improve microcirculation since vein and capillary thromboses are the main causes of tissue damage. Hyperbaric oxygen therapy may be used successfully in frostbite injuries. The immediate effect of HBOT is hyperoxygenation of ischemic tissues, resulting from increased amounts of dissolved oxygen in plasma directly in proportion to the partial pressure of inhaled oxygen. Hyperoxia may be of great benefit through numerous mechanisms: improvement of oxygen delivery and preservation of tissue viability in ischemic areas.^9^ The effect of HBOT on damaged tissues is complex. Hyperbaric oxygen therapy diminishes microorganism proliferation, activates antimicrobial agents, activates the immune system, and significantly improves pO_2_ in reversibly damaged peripheral tissues.^10^ Hyperbaric oxygen therapy could also be useful to prevent the late changes in the growing bones.^11^ Improvement in tissue survival following treatment of problem wounds and frostbite by hyperbaric oxygenation has been favorably demonstrated in published case reports. The first case of hyperbaric oxygen treatment of cold injury was reported by Ledingham^12^ and another 4 cases were reported by Ward et al^13^ in patients who had suffered frostbite during mountaineering expedition in the Alps. Folio et al^14^ reported a case of hand frostbite of a mountain climber who received HBOT. In another case report, Von Heimburg et al^11^ reported the potential therapeutic efficiency of HBOT in a boy with deep frostbite of both hands. Finderle and Cankar^15^ confirmed that HBOT is capable of improving nutritive skin blood flow in frostbitten areas. According to the Marx-schema for problem wounds, HBOT must be applied between 2 and 10 days after injury until the “pinking” appearance of the affected areas (2, 4 bar, total time at depth = 90 minutes, alternations of 100% O_2_, and air breathing). The increasing local tissue oxygen tension by HBOT improves and maintains the viability of the adjacent tissue. Therefore, vascular and cellular regeneration occurs faster and more efficiently.^16^,^17^

Prevention is the best strategy for reducing the morbidity and mortality due to frostbite. The first step is increasing the awareness of the employees and healthcare professionals about the risk of these injuries. Most of these injuries may be prevented by taking adequate precautions and education. Liquid helium cylinders should be used only in well-ventilated areas and in accordance with the manufacturer's instruction. These cylinders must always be kept in an upright position. Specialized hand trucks should be used for their movement. Workers should use sturdy work gloves, safety glasses with side shields, and safety shoes when handling compressed gas cylinders. In addition, the operation of transferring liquid helium requires at least 2 persons. Proper equipment must be used to protect the operators from frostbite.

In conclusion, the adjunctive HBOT is an alternative treatment of frostbite injuries for its contribution to healing, although it is still considered investigational. For the favorable outcomes of HBOT, it is better to start the treatment within the first 48 hours following injury. Hyperbaric oxygen therapy was effective in treatment against necrosis, infection, and tissue loss. However, controlled studies on the treatment of frostbite with HBOT are necessary to further establish the effectiveness of this treatment modality.
